# Health related quality of life in adult primary Ciliary dyskinesia patients in Cyprus: development and validation of the Greek version of the QOL-PCD questionnaire

**DOI:** 10.1186/s12955-020-01360-w

**Published:** 2020-04-22

**Authors:** Phivos Ioannou, Panayiotis Kouis, Maria G. Kakkoura, Margarita Kaliva, Aristoula Toliopoulou, Kyriacos Andreou, Laura Behan, Jane S. Lucas, Vicky Papanikolaou, George Charalambous, Nicos Middleton, Panayiotis K. Yiallouros

**Affiliations:** 1 Pediatric Pulmonology Unit, Hospital ‘Archbishop Makarios III’, Nicosia, Cyprus; 2 School of Health Sciences, Frederic University, Nicosia, Cyprus; 3 Respiratory Physiology Laboratory, Medical School, University of Cyprus, Nicosia, Cyprus; 4 Shakolas Educational Center of Clinical Medicine, Palaios Dromos Lefkosias-Lemesou 215/6,2029 Aglantzia, Nicosia, Cyprus; 5 Primary Ciliary Dyskinesia Centre, NIHR Southampton Respiratory Biomedical Research Unit, University of Southampton and University Hospital Southampton NHS Foundation Trust, Southampton, UK; 6 Department of Nursing, School of Health Sciences, Cyprus University of Technology, Limassol, Cyprus

**Keywords:** Primary Ciliary dyskinesia, Quality of life, Psychometric testing

## Abstract

**Background:**

The QOL-PCD questionnaire is a recently developed Health Related Quality of Life (HRQoL) instrument for Primary Ciliary Dyskinesia. The aim of this study was to translate the adult QOL-PCD questionnaire into Greek language and to conduct psychometric validation to assess its performance.

**Methods:**

Forward translations to Greek and backward translation to English were performed, followed by cognitive interviews in 12 adult PCD patients. The finalized translated version was administered to a consecutive sample of 31 adult, Greek speaking PCD patients in Cyprus for psychometric validation, which included assessment of internal consistency, test-retest reliability, construct and convergent validity. Internal consistency was assessed by Cronbach’s alpha test in terms of the overall and sub-scales. Test-retest reliability was assessed by repeat administration of the questionnaire within 2 weeks and calculation of the intra-class correlation (ICC). Construct validity was assessed by comparing different groups of patients based on a-priori hypotheses and convergent validity was evaluated by examining associations between the QOL-PCD and SF-36 questionnaires.

**Results:**

Moderate to good internal consistency was observed (Cronbach’s α: 0.46–0.88 across sub-scales) and test-retest reliability assessment demonstrated good repeatability for most scales (ICC: 0.67–0.91 across subscales). Patients of female gender, older age and lower lung function exhibited lower QOL-PCD scores in general, while high correlations for most QOL-PCD scales with corresponding SF-36 scales were observed, in particular for physical functioning (*r* = 0.78, *p* < 0.05).

**Conclusion:**

The adult version of QoL-PCD questionnaire has been translated according to international guidelines resulting to a cross-culturally validated Greek version which exhibited moderate to good metric properties in terms of internal consistency, stability, known-group and convergent validity.

## Introduction

Primary Ciliary Dyskinesia (PCD) is a rare, genetically heterogeneous disease characterized by dysfunction of motile cilia and disruption of mucociliary clearance. PCD patients usually suffer from chronic recurrent respiratory infections, which lead to chronic destructive airway disease, progressive loss of lung function and structural damage of the airways (bronchiectasis). Other manifestations of PCD include chronic wet cough, rhinorrhea, nasal polyps, frequent ear infections and situs abnormalities [1].

PCD clinical manifestations [2] as well as the commonly recommended treatment modalities such as daily respiratory physiotherapy and frequent or daily antibiotic administration [3], may impact the perceived quality of life of PCD patients. Previous studies have demonstrated that PCD patients, as well as their family members, may experience significant psychological stress, emotional and social impacts [4, 5]. In an effort to capture and access the impact of PCD from the patient perspective, a Health Related Quality of Life questionnaire (QOL-PCD) has been recently developed for pediatric, teenager and adult PCD patients [6–9] Following a rigorous process, which involved literature reviews, expert panel discussions, evaluation of existing measures and cognitive interviews, the QOL-PCD questionnaires were developed and validated in the English language. Subsequently, these were translated into several languages including Greek [10–13]. The QOL-PCD questionnaire has four age-specific versions (adults ≥18 years, adolescents 13–17 years, children 6–12 years and a parent-proxy questionnaire for children 6–12 years).

The aim of this study was to investigate, for the first time, the metric properties of the Greek version of the adult QOL-PCD questionnaire among adult PCD patients in Cyprus.

## Material and methods

### QOL-PCD adult questionnaire

The Greek version of the adult QOL-PCD questionnaire (available in Supplementary File 1) includes 40 questions that compose ten sub-scales: Physical Functioning (*n* = 5), Vitality (*n* = 3), Emotional Functioning (*n* = 5), Health Perception (*n* = 4), Treatment Burden (*n* = 4), Upper Respiratory Symptoms (*n* = 4), Lower Respiratory Symptoms (*n* = 6), Role (*n* = 4) Social Functioning (*n* = 3), Hearing Symptoms (*n* = 2). Higher scores in each subscale represent increased HRQoL.

### Translation to Greek language and cross-cultural adaptation

**Fig. 1:**
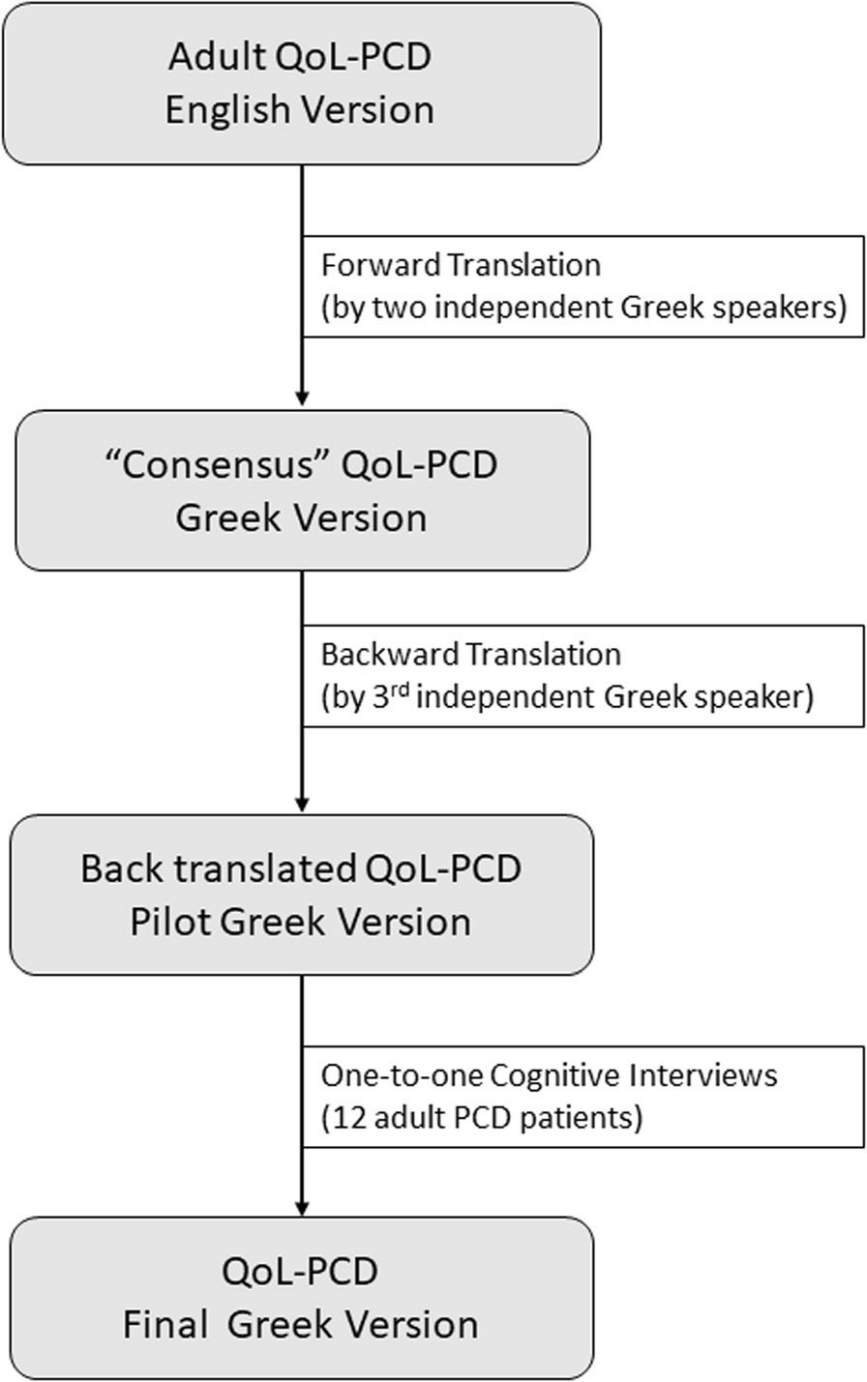
Schematic diagram of the translation process. The translation process included forward and backward translation by independent translators followed by one to one interviews with adult PCD patients to ensure that the questionnaire is comprehensive and culturally acceptable

The English version of the adult QOL-PCD questionnaire underwent forward translation by two independent translators (PK, AT) that were native Greek speakers with excellent command of the English language and good knowledge of PCD. Both independent translators, with the support of the team that developed the English version then agreed to a “consensus” forward translation that was subsequently back translated to English by a different translator who was familiar with PCD and had an excellent command of Greek (mother language) and English language. The back translated version of the Greek questionnaire was compared to the original English version of the questionnaire to check for differences. After careful review, the provisional version of the questions that constituted the Greek questionnaire was agreed through consensus. This step was followed by one-to-one cognitive interviews of 12 adult PCD patients. The cognitive interviews were performed by a respiratory nurse (PI), familiar with PCD and were followed by one-to-one, patient-interviewer discussions of comprehension issues and/or other obscure points. This process aimed to ensure the questionnaire is culturally acceptable, comprehensive and readable; subsequent minor modifications resulted in its final form. The Greek version of the questionnaire includes a total of 40 questions, similar to the English version. A schematic diagram of the translation process is available in Fig. 1.

### Validation study

#### Study population

Between January 2017 and June 2019, PCD patients attending the PCD clinic at Archbishop Makarios III hospital in Nicosia for their routine clinical evaluations, were asked to participate in the validation study by completing the QOL-PCD questionnaire if they fulfilled the following inclusion criteria: (1) “definite” or “highly likely” diagnosis of PCD according to the recent ERS guidelines for PCD diagnosis [14] (2) age > 18 years and (3) ability to speak and read Greek fluently. PCD diagnosis in Cyprus PCD Service relies on a combination of tests, including nasal Nitric Oxide (nNO), Transmission Electron Microscopy (TEM), High Speed Video Microscopy (HSVM) and/or identification of biallelic disease-causing mutations in one of the known PCD genes. The diagnostic subgroup “definite PCD” refers to patients with hallmark TEM findings and/or an identified biallelic PCD genetic mutation, while the diagnostic subgroup “highly likely PCD” refers to patients with abnormal HSVM findings and low nNO (using a cut-off of 77 nl/min as suggested by Leigh MW et al. 2013).

#### Clinical data

The anthropometric and clinical data of PCD patients are collected in a standardized format at each visit at the PCD clinic. Lung function assessments in terms of Forced Vital Capacity (FVC) and Forced Expiratory Volume in the first second (FEV_1_) are performed with a portable Spirometer (Vitalograph 2120) following the 2005 ERS/ATS recommendations [15] and sputum samples are collected at each visit for culture. FVC and FEV_1_ measurements were expressed as z-scores predicted for the patient’s age, sex and height [16].

#### Statistical analysis

The validation process of the Greek version of the adult QOL-PCD included assessment of the internal consistency, test-retest stability, construct and convergent validity for the whole questionnaire as well as for the different subscales of the questionnaire. Internal consistency of the QOL-PCD subscales were investigated by Cronbach’s α values. Cronbach’s α gives a score between 0 and 1, and Cronbach’s a values > 0.70 indicate good consistency of the questions that comprise the different subscales [17]. The repeatability of the QOL-PCD (test-retest) was assessed through a second, repeated administration of the questionnaire, a short interval (10–14 days) after completing the baseline assessment. The statistical intraclass correlation coefficient (ICC) criterion was applied to assess the stability of the QOL-PCD over this short time. This criterion gives values between − 1 and + 1 and values close to 1 indicate high repeatability of the questionnaire [18]. An ICC value > 0.75 demonstrates acceptable test-retest reliability across time [19]. Construct validity was examined against a-priori hypotheses. More specifically, we hypothesized that specific clinical features such as age, gender, FVC and FEV_1_ would correlate with specific scales. For example, it was anticipated that FEV_1_ would correlate with physical functioning, social functioning and lower respiratory symptoms. Age, FVC and FEV_1_ were treated as categorical values using the median as the cut-off value. The Mann-Whitney U test was used to compare levels of psychometric variables between the two groups.

To assess convergent validity, we examined the associations between the scales of QOL-PCD and the scales of the generic 36-Item Short Form (SF-36) HR-QoL questionnaire. Pearson correlation coefficients between scales of the two questionnaires with similar constructs were calculated. To interpret the correlation coefficients, we relied on Cohen’s guidelines which interpret correlations between 0.50 and 1.00 as strong, correlations between 0.30 and 0.50 as moderate, correlations between 0.10 and 0.30 as small and correlations < 0.1 as weak [20]. Post-hoc sample size calculations were carried out using the formula introduced by Bonnet et al. [21] for the evaluation of internal consistency (Cronbach’s α) across the scales of the QoL-PCD and using the G*power Software for the subgroup comparisons performed towards the assessment of construct validity [22].

#### Longitudinal construct validity

Towards assessing the longitudinal construct validity (responsiveness) of the tool, patients were asked to complete the QOL-PCD questionnaire during their routine visits to the PCD clinic for a total follow-up period of 30 months. The changes in QOL-PCD Physical Functioning and Lower Respiratory Symptoms score between two consecutive follow-up visits were plotted against the corresponding changes in FEV_1_ and FVC, while the relationship was assessed using linear regression analysis.

Statistical analysis was conducted with the Statistical Package of Social Sciences IBM SPSS 22 and *p* < 0.05 was considered to be statistically significant.

## Results

### Study participants

A total of fifty-two (52) patients were followed up at the PCD clinic in Archbishop Makarios III during the period 2017–2019. Thirty-one (31) patients older than 18 years old agreed to take part in the study. Of them, 18/31 (58.1%) were female and 14/31 (45.2%) had Situs Inversus. The median age of the patients at the time of the study was 33.6 and the median age of patient presentation to our center was 23.8. All participants were Greek-speakers and 28/31 (90.3%) were of Greek-Cypriot ethnicity, 2/31 (6.5%) of Arab ethnicity and 1/31 (3.2%) of British ethnicity. Table 1 describes the demographic, diagnostic and clinical characteristics of the participants.

**Table 1:** Demographic, diagnostic and clinical characteristics of adult patients participating in the study

Parameter	Adult PCD patients (***n*** = 31)	
***Demographic information***		
Current Age^a^	33.6 (22.2–50.9)	
Gender (female)	18/31	58.1%
Age at Presentation^a^	23.79 (17.8–45.1)	
Situs Abnormalities	14/31	45.2%
***Diagnostic characteristics***		
Nasal Nitric Oxide (nl/min)^a^	24.9 (13.0–48.0)	
TEM result		
Normal TEM	8/31	25.8%
ODA + IDA	9/31	29.0%
ODA only	6/31	19.4%
CP/ IDA + MD	8/31	25.8%
Other	0/31	0%
HSVM result		
Normal HSVM	0/31	0%
Immotile/Almost Immotile	13/31	41.9%
Extremely stiff due to reduced ciliary bending	8/31	25.8%
Stiff Beating pattern	3/31	9.7%
Circular Pattern	7/31	22.6%
Other	0/31	0%
***Clinical Characteristics***		
Chronic Rhinorrhoea	31/31	100%
Chronic wet cough	31/31	100%
History of NRDS	14/31	45.2%
History of nasal polyps	8/30	26.7%
History of Pneumonia	12/29	41.4%
History of Heamoptysis	3/31	9.7%
History of lung resection	5/31	16.1%

^a^Median and Interquartile Range *TEM* Transmission Electron Microscopy, *HSVM* High Speed Video Microscopy, *ODA + IDA* Combined Outer Dynein Arm defect and Inner Dynein Arm defect, *ODA* Isolated Outer Dynein Arm defect, *CP* Central Pair defect, *IDA + MD* Inner Dynein Arm and Microtubular Disorganisation defect, *NRDS* Neonatal Respiratory Distress Syndrome

### Internal consistency and test-retest reliability

Overall, most QOL-PCD subscales demonstrated moderate to strong internal consistency (Cronbach’s α: 0.46–0.88 across subscales). The lowest internal consistency was observed for Vitality (Cronbach’s α: 0.46) while the highest was observed for Physical Functioning (Cronbach’s α: 0.88). All participants completed a second, repeated administration of the questionnaire within 2 weeks after completing the baseline assessment and test-retest reliability assessment demonstrated good repeatability for most scales (ICC: 0.67–0.91 across subscales). The results for internal consistency and test-retest reliability assessment are summarized in Table 2.

**Table 2:** Internal consistency of QoL-PCD scales measured by Cronbach’s α and test–retest reliability measured by ICC

Scale	# Items	Scale Median(IQR)	Cronbach’s α	ICC (95%)
**Physical Functioning**	5	80.0 (46.7–93.3)	0.880	0.910 (0.803–0.959)
**Vitality**	3	66.7 (44.4–77.8)	0.463	0.817 (0.598–0.916)
**Emotional Functioning**	5	86.7 (63.7–93.3)	0.642	0.893 (0.766–0.951)
**Treatment Burden**	4	66.7 (41.7–83.3)	0.809	0.670 (0.167–0.869)
**Social Functioning**	3	33.3 (0.0–66.7)	0.681	0.768 (0.491–0.894)
**Role**	4	66.7 (58.3–83.3)	0.542	0.902 (0.785–0.955)
**Health Perspective**	4	50.0 (41.7–66.7)	0.639	0.670 (0.277–0.850)
**Upper Respiratory Symptoms**	4	58.3 (41.7–83.3)	0.687	0.853 (0.678–0.933)
**Lower Respiratory Symptoms**	6	61.1 (44.4–72.2)	0.784	0.895 (0.769–0.952)
**Hearing Symptoms**	2	66.7 (58.3–100)	0.661	0.838 (0.645–0.926)

### Construct validity

Based on prior hypotheses, patients were stratified by the following variables that were anticipated to be associated with HRQoL: Sex (male-female), Age (defined by the median value of 33.6 years), FVC z-score (defined by the median value of − 1.47) and FEV_1_ z-score (defined by the median value of − 2.00). Females, patients > 33.6 years old and patients with lower lung function tended to have lower QOL-PCD scores in general, although in most comparisons statistical significance was not achieved. More specifically, for physical functioning subscale, the median score was 86.7 (IQR: 60.0–93.3) for males and 60.0 (IQR: 38.3–88.3) for females, (p_value_ for the difference in medians = 0.060). Similarly, the median physical functioning score for older patients was lower (60.0, IQR: 40.0–93.3) compared to younger patients (80.0, IQR: 54.9–93.3) (p_value_ = 0.280). As hypothesized, the median physical functioning score in patients with FEV_1_ z-score < 2.00 (56.7, IQR: 34.9–80.0) was also lower compared to the median score in patients with FEV_1_ z-score **≥** 2.00 (93.3, IQR: 66.7–93.3, *p*_value_ = 0.023). Similarly, the median score in patients with FVC z-score < 1.47 (60.0, IQR: 33.3–80.0) tended to be lower than in patients with higher FVC z-score (86.7, IQR: 48.3–93.3), (*p*_value_ = 0.110). Significant differences were also observed between younger and older patients in terms of Social Functioning (50.0, IQR: 33.3–66.7 Vs 22.2, IQR: 0.0–44.4, *p*_value_ = 0.022) and upper respiratory symptoms 66.7, IQR: 52.1–83.3 Vs 58.3, IQR: 33.3–66.7, *p*_value_ = 0.042 as well as between high and low FEV_1_ z-scores and the subscale for Lower Respiratory Symptoms (66.7, IQR: 61.1–77.8 Vs 50.0, IQR: 38.9–65.3, *p*_value_ = 0.016). Table 3 presents the comparisons of Physical Functioning, Social Functioning and Lower Respiratory Symptoms with Gender, Age, FEV_1_ and FVC and Supplementary File 2 presents the results for all QoL-PCD subscales.

**Table 3:** Construct validity of QoL-PCD assessed through the association of specific QoL-PCD scale values with Gender, Age, FEV1 and FVC

Parameter	Physical Functioning		Social Functioning		Lower Respiratory Symptoms	
	Value	Sig.	Value	Sig.	Value	Sig.
**Male**	86.7 (60.0–93.3)	0.060	44.4 (11.1–66.7)	0.395	66.7 (52.8–75.0)	0.258
**Female**	60.00 (38.3–88.3)		27.8 (0.00–55.6)		61.1 (37.5–68.1)	
**< 33.6 years**^**a**^	80.00 (54.9–93.3)	0.280	50.00 (33.3–66.7)	0.022	63.9 (45.8–76.4)	0.232
**> 33.6 years**	60.0 (40.0–93.3)		22.2 (0.00–44.4)		61.10 (33.3–66.7)	
**<−2 FEV1**^**a**^**z-score**	56.7 (34.9–80.0)	0.023	27.8 (0.00–52.80)	0.316	50.00 (38.9–65.3)	0.016
**≥ − 2 FEV1 z-score**	93.3 (66.7–93.3)		44.4 (22.2–66.7)		66.7 (61.1–77.8)	
**<−1.47 FVC**^**a**^**z-score**	60.00 (33.3–80.0)	0.110	31.3 (0.0–55.6)	0.367	55.6 (38.9–66.7)	0.112
**≥ − 1.47 FVC z-score**	86.7 (48.3–93.3)		38.8 (13.9–66.7)		66.70 (51.4–77.8)	

^**a**^Median Age, Median FEV1 Z-Score, Median FVC Z-score

### Convergent validity

Strong correlations were observed between similar constructs of QOL-PCD and the SF-36 in the scales of physical functioning (*r* = 0.776, *p* < 0.05), vitality (*r* = 0.703, *p* < 0.05) and emotional functioning/mental health (*r* = 0.706 *p* < 0.05). Upper respiratory and lower respiratory symptoms were correlated with all SF-36 scales. Table 4 presents all correlations between QOL-PCD scales and SF-36.

**Table 4:** Correlation coefficients between scales from QoL-PCD and the scales of the generic SF-36 HRQoL questionnaire

	SF36 Physical Functioning	SF36 Role Physical	SF36 Bodily Pain	SF36 General Health	SF36 Vitality	SF36 Social Functioning	SF36 Role Emotional	SF36 Mental Health
**Physical Functioning**	0.776*	0.645*	0.351	0.407*	0.665*	0.650	0.605*	0.648*
**Vitality**	0.804*	0.696*	0.541*	0.298	0.703*	0.710	0.733*	0.588*
**Emotional Functioning**	0.675*	0.475*	0.406*	0.333	0.711*	0.621	0.530*	0.706*
**Treatment Burden**	0.113	−0.077	0.013	−0.061	−0.015	−0.050	0.066	0.033
**Role**	0.509*	0.469*	0.495*	0.288	0.460*	0.497*	0.403	0.403
**Social Functioning**	0.325	0.240	0.251	0.132	0.175	0.238	0.157	0.224
**Health Perspective**	0.307	0.209	0.268	0.461*	0.244	0.190	0.084	0.212
**Upper Respiratory**	0.534*	0.577*	0.352	0.478*	0.521*	0.489*	0.401	0.485*
**Lower Respiratory**	0.619*	0.644*	0.649*	0.635*	0.682*	0.571*	0.519	0.706*
**Hearing Symptoms**	0.226	0.295	0.207	0.291	0.168	0.180	0.241	0.034

*Significant at the 0.05 confidence level, Highlighted cells correspond to a-priori assumed positive relationships

### Longitudinal construct validity

**Fig. 2:**
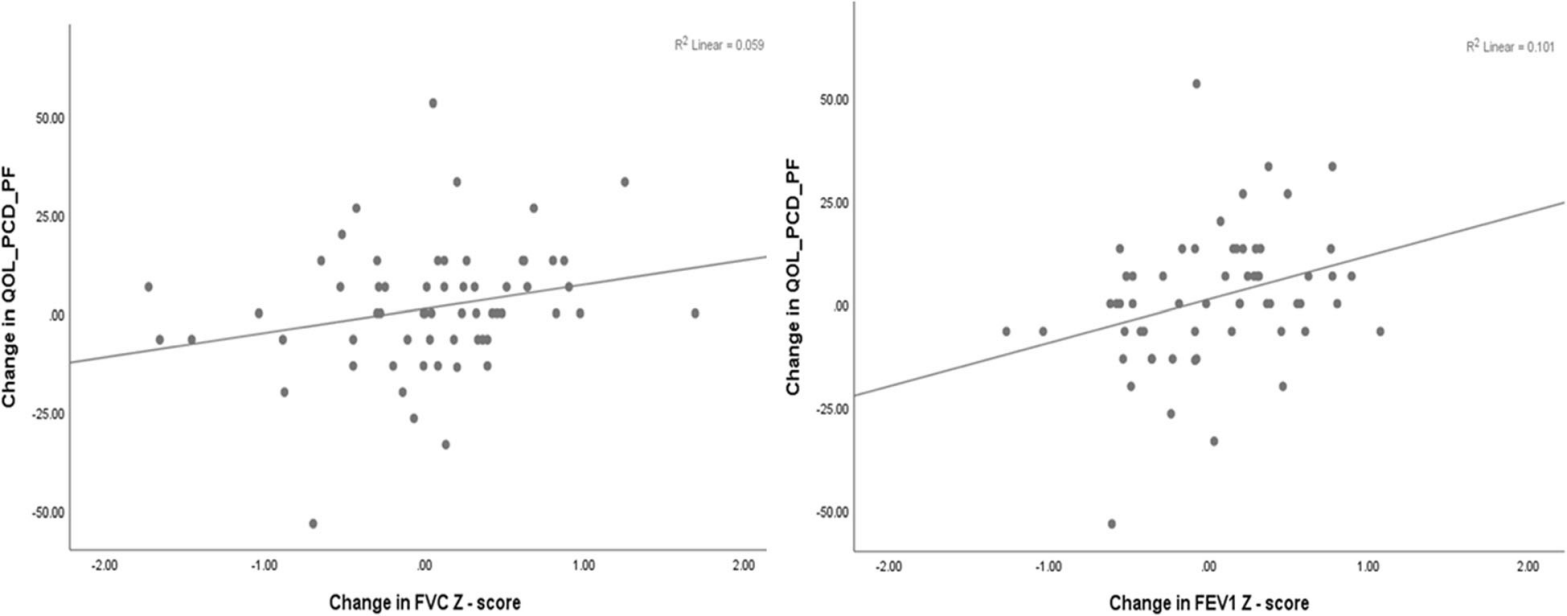
Association of FEV1 and FVC z-score changes with Physical Functioning subscale changes. Scatter-plots of FEV1 and FVC z-score between consecutive clinic visits and corresponding Physical Functioning subscale changes

There was a weak association between lung function indices (FEV_1_ and FVC) and the QOL-PCD subscale Physical Functioning. The scatter-plots of FEV_1_ and FVC z-score changes between consecutive clinic visits and corresponding Physical Functioning subscale changes are presented in Fig. 2. A unit increase in FVC z-score was associated with a 6.15 (95% CI: − 0.24 – 12.54) points increase in the QOL-PCD subscale (p_value_ = 0.059). Similarly, a unit increase in FEV_1_ z-score was associated with a 10.55 (95% CI: 2.26–18.83) points increase in the QOL-PCD subscale (*p*_value_ = 0.013). The relationships between Lower Respiratory Symptoms and FVC z score (β = 0.973, 95% CI: − 6.28 – 8.23, *p*_value_ = 0.789) and FEV_1_ z-score (β = − 5.94, 95% CI: − 15.32 – 3.45, *p*_value_ = 0.211) were not significant.

## Discussion

We present the translation and validation of the adult QOL-PCD questionnaire into the Greek language and its administration among adult PCD patients in Cyprus. The psychometric validation of the questionnaire demonstrated that it is a robust and valid instrument to measure HRQoL in PCD patients. Overall, the Greek version of the adult QOL-PCD questionnaire was characterised by moderate to good internal consistency across subscales (Cronbach’s a range: 0.463–0.880) and excellent test–retest reliability across all subscales within a two-week period (ICC range: 0.67–0.91). Compared to generic HRQoL tools such as the SF-36, moderate to high correlations were observed for subscales that were measuring the same constructs such as Physical Functioning, Emotional Functioning and Vitality. In relation to the English version of the QOL-PCD questionnaire, we observed similar psychometric properties, especially regarding the Physical Functioning subscale which demonstrated the greatest internal consistency and test-retest reliability in both validation studies. Similarly, convergent validity between subscales of the QOL-PCD and SF-36 subscales was consistent in both validation studies. In particular, subscales such as Physical Functioning, Mental health and Vitality demonstrated significant correlations, while the subscale on Social Functioning demonstrated a non-significant correlation in both studies. In contrast to the English version, the Vitality subscale was characterised by low internal consistency (Greek version Cronbach’s a: 0.463 Vs English version Cronbach’s a: 0.79). Low internal consistency was driven by item #7: “You felt energetic”, which was poorly correlated with the other two items that were part of the Vitality subscale (item #5: You felt tired” and item #8: “You felt exhausted”. Exclusion of item #7 led to a Cronbach’s a equal to 0.738 for the Vitality subscale.

The vast majority of PCD patients suffer from year round rhinorrhea, nasal congestion as well as chronic wet cough. Furthermore, compromised mucociliary clearance frequently results in recurrent lower (pneumonia) and upper respiratory infections (rhinosinusitis). Upper airway manifestations also include nasal polyps, serous otitis and impaired hearing [2]. The impact of these manifastations on PCD patients, have been assessed previously using non-PCD specific HRQoL tools such as the St. George’s Respiratory Questionnaire (SGRQ) and the SF-36 questionnaire, as well as by using qualitative approaches. A recent systematic review summarized the results of these early studies and highlighted physical, emotional and social functioning as the main areas characterizing the impact of PCD [23].

Previous studies in patients with chronic respiratory diseases indicated that factors such as age, overall lung function and gender may influence HRQoL. In Cystic Fibrosis (CF), age has been shown to have a strong association with HRQoL subscales such as physical functioning, vitality and health perception [24]. A similar decline in several scales of the SGRQ with age was also observed in cross-sectional surveys of PCD patients in the UK [25] and Italy [26], eventually leading to significantly lower HRQoL compared to local general population standards. In our adult PCD population, social functioning, vitality, upper respiratory symptoms and physical functioning median scores tended to be lower in the older compared to younger patients, although this difference did not reach statistical significance in the latter subscale.

Female gender is a well-known predictor of adverse disease course in CF [27, 28] and has been recently proposed as a predictor of poor lung function in PCD [29]. HRQoL scores in CF females have been shown to be lower in subscales such as emotional functioning, social functioning and respiratory symptoms in some studies but not in others [24]. In terms of physical functioning, female CF patients are known to demonstrate lower scores in comparison to male patients, even after adjustments for other factors such as FEV_1_, age and frequency of pulmonary exacerbations [30, 31]. In line with these findings, we demonstrated a tendency for increased disease burden among our female PCD patients especially relating to physical functioning and emotional functioning.

Previous CF studies also demonstrated that poorer lung function is associated with worse physical HRQoL, both cross-sectionally [32] and longitudinally [33]. In particular, FEV_1_ was shown to be consistently associated with physical functioning and other HRQoL scales (at a lesser degree) across several studies involving adolescent CF patients [24]. The relationship between FEV_1_ and physical functioning was also observed among adult PCD patients with mean scores of 41.8 and 80.5 in patients with FEV_1_ < 40 and > 75% respectively [8]. In our adult PCD population the median scores for physical functioning and lower respiratory symptoms in patients with FEV_1_ z-score < 2.00 Vs FEV_1_ z-score ≥ 2.00 were significantly lower, confirming the increasing burden of the disease with progression of loss of lung function. Interestingly, during the 30-month follow-up period, a weak association between changes in lung function and changes in physical functioning between clinical visits was demonstrated. However, this association was not evident for Lower Respiratory Symptoms, suggesting that lung function may not be a sensitive outcome measure to capture changes in disease burden in PCD. This was also evident in the validation study of the original English version of the adult QOL-PCD where significant associations between FEV_1_ and physical functioning were observed in the cross-sectional analysis but not for Lower Respiratory Symptoms [8]. In addition, lack of standardised, national lung function reference values may further limit the sensitivity of spirometry to capture the variability in PCD disease burden. Perhaps a more sensitive indicator of lung function such as the lung clearance index could be an alternative outcome measure for short term and long-term changes in PCD disease severity [34]. Lastly, cultural differences have been found to influence the self-perceived quality of life in previous studies [35] but given the almost completely homogeneous ethnic profile in our cohort, the probability of confounding due to cultural differences is limited.

This validation study of the Greek version of the QOL-PCD benefited from a clearly defined protocol that was recently applied in a multicentre setting during the validation of the English version of the QOL-PCD questionnaire and a continuous 30-month follow-up period of the participants. In addition, participants were cases that received a “definite” or “highly likely” diagnosis of PCD according to the ERS diagnostic guidelines ensuring diagnostic certainty in a disease that is known to be quite heterogeneous and difficult to diagnose. Nevertheless, as PCD is a rare disease, the small sample size remains an important limitation of this study. The small number of participants was not though a result of non-response or exclusion of participants as almost all known adult PCD patients in Cyprus took part in the study. In addition, post-hoc sample size calculations demonstrated that for the evaluation of internal consistency, the minimum sample size required was 20 subjects while for the subgroup analyses the minimum sample size required was 28. All of the above confirm that the sample was reliable, representative and reduces selection bias. Furthermore, although low sample size may have resulted in increased error margins in this study, it is noteworthy that our findings are in line with the findings of the adult QOL-PCD validation in English language that enrolled a total of 72 patients.

Lastly, this work did not examine the effect of pulmonary exacerbations on HRQoL in PCD as a formal definition for pulmonary exacerbation in PCD was not available at the beginning of this study. Since 2019, an expert consensus statement has been proposed for PCD [36] and future studies are expected to focus on the responsiveness of QOL-PCD to pulmonary exacerbations in PCD.

## Conclusions

QOL-PCD is the first disease-specific, health related quality of life questionnaire for PCD. The adult version of QoL-PCD has been translated according to international guidelines resulting in a cross-culturally validated Greek version which exhibited moderate to good metric properties in terms of internal consistency, stability, known-group and convergent validity. The translation and validation of the QOL-PCD across different languages will allow the consistent assessment of HRQoL in different countries and across a larger number of PCD patients. Improved understanding of PCD impact and the use of HRQoL as a valid outcome measure in clinical trials and clinical practice across the world will lead to improved care for PCD patients and their families.

## Supplementary information

**Additional file 1.**

**Additional file 2: Table S1.** Construct validity of QoL-PCD assessed through the association of all QoL-PCD scale values with Gender, Age, FEV1 and FVC.

## Data Availability

The datasets used and analyzed during the current study are available from the corresponding author on reasonable request.

## Abbreviations

HRQoL: Health Related Quality of Life
PCD: Primary Ciliary Dyskinesia
ICC: Intra-class correlation
nNO: Nasal nitric Oxide
TEM: Transmission Electron Microscopy
HSVM: High Speed Video Microscopy
FVC: Forced Vital Capacity
FEV1: Forced Expiratory Volume in the first second
ERS: European Respiratory Society
ATS: American Thoracic Society
SF-36: 36-Item Short Form questionnaire
