# Supplementary material for: Health related quality of life in adult primary Ciliary dyskinesia patients in Cyprus: development and validation of the Greek version of the QOL-PCD questionnaire
Source: Health Qual Life Outcomes. 2020 Apr 22;18:105. doi: 10.1186/s12955-020-01360-w (PMC7178983; doi:10.1186/s12955-020-01360-w)
Supplement: Supplementary file 1 — Additional file 1. [file 12955_2020_1360_MOESM1_ESM.pdf]

ΠΔΚ ΕΡΩΤΗΜΑΤΟΛΟΓΙΟ ΠΟΙΟΤΗΤΑΣ ΖΩΗΣ

Ενήλικες Ηλικίας 18 ετών και άνω

Η κατανόηση των επιπτώσεων της ασθένειας και των θεραπειών που λαμβάνεις στην καθημερινή σου ζωή, μπορεί να βοηθήσει την θεραπευτική σου ομάδα να παρακολουθεί την πορεία της υγείας σου και ανάλογα να προσαρμόζει τις θεραπείες σου. Για τον σκοπό αυτό, το συγκεκριμένο ερωτηματολόγιο ποιότητας ζωής σχεδιάστηκε συγκεγκριμένα για άτομα με ΠΔΚ. Ευχαριστούμε για την προθυμία σου να συμπληρώσεις αυτό το ερωτηματολόγιο.

Οδηγίες: Οι ακόλουθες ερωτήσεις αφορούν την παρούσα κατάσταση της υγείας σου, όπως την αντιλαμβάνεσαι. Αυτές οι πληροφορίες θα μας επιτρέψουν να κατανοήσουμε καλύτερα πως αισθάνεσαι στην καθημερινή σου ζωή.
Παρακαλώ απαντήσε όλες τις ερωτήσεις. **Δεν υπάρχουν** σωστές οι λανθασμένες απαντήσεις! Εαν δεν είσαι σίγουρος πως να απαντήσεις, επίλεξε την απάντηση που ανταποκρίνεται καλύτερα στην δική σου κατάσταση.

Σημείωση για ιατρούς και ερευνητές:

Αυτό το ερωτηματολόγιο μπορεί να χρησιμοποιηθεί με τη σύμφωνο γνώμη της ομάδας των δημιουργών του. Οι δημιουργοί ζητούν όπως τα δεδομένα που θα συλλεχθούν από την χρήση των ερωτηματολογίων, χρησιμοποιηθούν για την περαιτέρω εγκυροποίηση του. Δεδομένα που έχουν σχέση με αυτό το ερωτηματολόγιο δεν μπορούν να χρησιμοποιηθούν πρίν από την δημοσίευση των αποτελεσμάτων της μελέτης εγκυροποίησης που διεξήχθει από την ομάδα των δημιουργών, κάτι που αναμένεται να γίνει το Φθινόπωρο του 2016.

Για περισσότερες πληροφορίες και άδειες χρήσης παρακαλώ επικοινωνήστε:

- Για Βόρεια Αμερική: Professor Alexandra Quittner, email: aquittner@miami.edu
- Για Ευρώπη: Professor Jane Lucas, email: jlucas1@soton.ac.uk

©2015 Lucas, Leigh & Quittner

ΠΔΚ ΕΡΩΤΗΜΑΤΟΛΟΓΙΟ ΠΟΙΟΤΗΤΑΣ ΖΩΗΣ **Ενήλικες Ηλικίας 18 ετών και άνω**

Μέρος Ι. Ποιότητα Ζωής

Παρακαλώ σημειώσε (✓)στο κουτάκι που αντιστοιχεί στην απάντηση σου.

Κατά την διάρκεια της περασμένης βδομάδας, σε ποιο βαθμό δυσκολεύτηκες:	**Αρκετή δυσκολία**	**Κάποια δυσκολία**	**Λίγη δυσκολία**	**Καμία δυσκολία**
**1.** Να εκτελείς έντονες δραστηριότητες (οπως κηπουρική ή άσκηση)	☐	☐	☐	☐
**2.** Να περπατάς το ίδιο γρήγορα με τους άλλους	☐	☐	☐	☐
**3.** Να ανέβεις από τις σκάλες περισσότερους από ένα ορόφους	☐	☐	☐	☐
**4.** Να κουβαλάς βαριά αντικείμενα, όπως βιβλία και τσάντες με ψώνια	☐	☐	☐	☐

Κατά την διάρκεια της περασμένης βδομάδας, *σημείωσε πόσο συχνά:*	**Πάντα**	**Συχνά**	**Μερικές φορές**	**Ποτέ**
**5.** Ένιωθες κουρασμένος/η..	☐	☐	☐	☐
**6.** Ένιωθες ανήσυχος/η	☐	☐	☐	☐
**7.** Ένιωθες ενεργητικός/ή..	☐	☐	☐	☐
**8.** Ένιωθες εξουθενωμένος/η	☐	☐	☐	☐
**9.** Ένιωθες λυπημένος/η...	☐	☐	☐	☐
**10.** Ένιωθες κατάθλιψη..	☐	☐	☐	☐

Παρακαλώ σημείωσε (✓)στο κουτάκι που αντιστοιχεί στην απάντηση σου.

Βρίσκεσαι στο παρόν στάδιο σε θεραπεία (φαρμακευτική αγωγή από το στόμα ή εισπνεόμενη, φυσιοθεραπεία/καθαρισμό αεραγωγών);

☐***Ναι*** ☐***Όχι (συνέχισε στο ερώτημα 17)***

Κατά την διάρκεια της περασμένης βδομάδας, σημείωσε:

11. Πόσο χρόνο ξοδεύεις κάθε μέρα για τις θεραπείες για την ΠΔΚ;

a. Πολύ
b. Μέτριο
c. Λίγο
d. Σχεδόν καθόλου

Κατά την διάρκεια της περασμένης βδομάδας, *σημειώστε σε ποιο βαθμό:*	**Καθόλου**	**Λίγο**	**Μέτρια**	**Πολύ**
**12.** Οι θεραπείες για την ΠΔΚ, έκαναν την καθημερινή σου ζωή πιο δύσκολη	☐	☐	☐	☐
**13.** Ήταν δύσκολο να ενσωματώσεις τις θεραπείες για την ΠΔΚσε καθημερινή βάση..	☐	☐	☐	☐

©2015 Lucas, Leigh & Quittner

**14.** Η φυσιοθεραπεία/καθαρση αεραγωγών σε έκανε να αισθάνεσαι πιο γρήγορα κουρασμένος στην καθημερινή σου ζωή	☐	☐	☐	☐

Σκεφτόμενος την υγεία σου κατα την διάρκεια της περασμένης βδομάδας, σημείωσε σε ποιο βαθμό η κάθε πρόταση ισχύει για εσένα.

	**Πολύ αληθές**	**Κυρίως αληθές**	**Λίγο αληθές**	**Καθόλου αληθές**	**Δεν ισχύει**
**15.** Νιώθω άνετα να βήχω μπροστά σε άλλους..	☐	☐	☐	☐	
**16.** Ανησυχώ μήπως έλθω σε έπαφη σε άλλους που είναι άρωστοι..........	☐	☐	☐	☐	
**17.** Αισθάνομαι μοναξιά..	☐	☐	☐	☐	
**18.** Νιώθω άνετα να φυσώ την μύτη μου μπροστά σε άλλους	☐	☐	☐	☐	
**19.** Αισθάνομαι άνετα να κάνω θεραπεία (κάθαρση αεραγωγών/ φυσιοθεραπεία) μπροστά σε άλλους..	☐	☐	☐	☐	☐

Μέρος ΙΙ. Σχολείο, Δουλειά, ή Καθημερινές Δραστηριότητες

Κατά την διάρκεια της περασμένης βδομάδας:

20. Σε ποιο βαθμό είχες δυσκολία να ανταποκριθείς στις ανάγκες της δουλειάς σου, τις οικιακές εργασίες ή άλλες καθημερινές δραστηριότητες;

a. Δεν είχες πρόβλημα να ανταποκριθείς
b. Κατάφερνες να ανταποκριθείς αλλά με δυσκολία.
c. Έμενες πίσω
d. Δεν κατάφερες να κάνεις αυτές τις δραστηριότητες καθόλου

21. Σε ποιο βαθμό, η ΠΔΚ επηρέαζει την εκπλήρωση των στόχων που έχεις στην δουλειά στο σπίτι, στην οικογένεια ή στην προσωπική σου ζωή;

☐ Πολύ ☐ Μέτρια ☐ Λίγο ☐ Καθόλου

Σκεφτόμενος την υγεία σου την ***περασμένη βδομάδα****, σημειώσε σε ποιο βαθμό ισχυεί η κάθε πρόταση για σένα.*

	**Πολύ αληθές**	**Κυρίως αληθές**	**Λίγο αληθές**	**Καθόλου αληθές**
**22.** Είναι δύσκολο να σχεδιάζω για το μέλλον (π.χ. αλλαγή δουλειών ή να κάνω οικογένεια)	☐	☐	☐	☐
**23.** Έχω μία φυσιολογική ζωή	☐	☐	☐	☐
**24.** Ανησυχώ μήπως η υγεία μου χειροτερεύσει..	☐	☐	☐	☐
**25.** Αισθάνομαι υγιής..	☐	☐	☐	☐
**26.** Καταφέρνω να ενσωματώνω σε καθημερινή βάση τις θεραπείες για την ΠΔΚ...	☐	☐	☐	☐

©2015 Lucas, Leigh & Quittner

Παρακαλώ σημείώσε (✓)στο κουτάκι που αντιστοιχεί στην απάντηση σου.

27. Πως νομίζεις ότι είναι η υγεία σου τώρα;

a. Άριστη
b. Καλή
c. Μέτρια
d. Κακή

Μέρος III. Συμπτώματα

Παρακαλώ σημείωσε (✓)στο κουτάκι που αντιστοιχεί στην απάντηση σου.

*Σημείωσε πως αισθανόσουν κατα την διάρκεια της περασμένης **βδομάδας**.*		Σε μεγάλο βαθμό	Μερικώς	Λίγο	Καθόλου
**28.**	Αισθανόσουν συμφόρηση στο στήθος;..	☐	☐	☐	☐
**29.**	Είχες πρόβλημα να ακούς καθαρά (εάν φοράς ακουστικά: είχες πρόβλημα να ακούς καθαρά χωρίς τα ακουστικά)	☐	☐	☐	☐
**30.**	Ένιωθες την μύτη σου να στάζει υγρό προς στον λαιμό σου (είχες δηλαδή εσωτερική ρινική καταρροή)	☐	☐	☐	☐
**31.**	Είχες πόνο στα ιγμόρεια (στο πρόσωπο ή στο κεφάλι)	☐	☐	☐	☐
**32.**	Είχες σφίξιμο στο στήθος	☐	☐	☐	☐
**33.**	Αναγκαζόσουν να βήχεις φλέγματα	☐	☐	☐	☐

Εάν η απάντηση σας είναι Καθόλου συνεχιστε στην ερώτηση 35

Κατά την διάρκεια της περασμένης βδομάδας:

34. Το φλέγμα σου ήταν κυρίως ☐Καθαρό ☐Καθαρό προς κίτρινο ☐Κιτρινοπράσινο ☐Πρασινωπό ☐Πράσινο με ίχνη αίματος ☐Δεν γνωρίζω

*Σημείωσε πόσο συχνά κατα την προηγούμενη **βδομάδα**:*		Πάντα	Συχνά	Μερικές φορές	Ποτέ
**35.**	Είχες δύσπνοια σε περιπτώσης πιο έντονης δραστηριότητας (π.χ. οικιακές εργασίες ή κηπουρική)	☐	☐	☐	☐
**36.**	Είχες συριγμό όταν ανάπνεες...	☐	☐	☐	☐
**37.**	Είχες ρινική καταρροή	☐	☐	☐	☐

©2015 Lucas, Leigh & Quittner

*Σημείωσε πόσο συχνά κατά την περασμένη **βδομάδα:***	**Πάντα**	**Συχνά**	**Μερικές φορές**	**Ποτέ**
**38.** Είχες δύσπνοια όταν μιλούσες	☐	☐	☐	☐
39. Ένιωθες τα αυτιά σου βουλωμένα	☐	☐	☐	☐
**40.** Είχες μπλοκαρισμένη μύτη	☐	☐	☐	☐

Παρακαλώ βεβαιώσου ότι απάντησες όλες τις ερωτήσεις.

ΣΕ ΕΥΧΑΡΙΣΤΟΥΜΕ

©2015 Lucas, Leigh & Quittner
