# Supplementary material for: Health related quality of life in adult primary Ciliary dyskinesia patients in Cyprus: development and validation of the Greek version of the QOL-PCD questionnaire
Source: Health Qual Life Outcomes. 2020 Apr 22;18:105. doi: 10.1186/s12955-020-01360-w (PMC7178983; doi:10.1186/s12955-020-01360-w)
Supplement: Supplementary file 2 — Additional file 2: Table S1. Construct validity of QoL-PCD assessed through the association of all QoL-PCD scale values with Gender, Age, FEV1 and FVC. [file 12955_2020_1360_MOESM2_ESM.docx]

**Supplementary Table 1:** Construct validity of QoL-PCD assessed through the association of all QoL-PCD scale values with Gender, Age, FEV1 and FVC.

|  | **Gender** | | | **Age (years)** | | | **FEV1 (z-score)** | | | **FVC (z-score)** | | |
| --- | --- | --- | --- | --- | --- | --- | --- | --- | --- | --- | --- | --- |
| **Variable** | **Males**  **(n=13)** | **Females**  **(n=18)** | **P value** | **< 33.6** | **≥ 33.6** | **P value** | **<-2.00**  **z-score** | **≥ -2.00**  **z-score** | **P value** | **<-1.47 FVC Z score** | **≥ -1.47 FVC Z score** | **P value** |
| **Physical Functioning** | 86.7  (60.0-93.3) | 60.0  (38.3-88.3) | 0.060 | 80.0  (54.9-93.3) | 60.0  (40.0-93.3) | 0.280 | 60.0  (33.3-80.0) | 90.0  (56.7-93.3) | 0.023 | 60.00  (33.3-80.0) | 86.7  (48.3-93.3) | 0.110 |
| **Vitality** | 66.7  (50.0-77.8) | 66.7  (41.7-77.8) | 0.499 | 66.7  (58.3-86.1) | 55.6  (33.3-66.7) | 0.036 | 66.7  (44.4-77.8) | 66.7  (55.6-77.8) | 0.467 | 66.7  (44.4-77.8) | 66.7  (47.2-77.8) | 0.762 |
| **Emotional Functioning** | 93.3  (80.0-93.3) | 73.3  (56.7-88.3) | 0.043 | 86.7  (63.3-93.3) | 80.0  (66.7-93.3) | 0.793 | 73.3  (46.7-93.3) | 86.7  (74.9-93.3) | 0.130 | 86.7  (46.7-93.3) | 83.3  (68.3-93.3) | 0.432 |
| **Treatment Burden** | 70.9  (58.3-95.8) | 58.3  (41.7-83.3) | 0.434 | 66.7  (45.8-79.2) | 66.7  (41.7-91.7) | 0.899 | 75.0  (58.3-87.5) | 62.5  (35.5-79.2) | 0.416 | 75.0  (58.3-85.4) | 58.3  (29.2-83.4) | 0.324 |
| **Role** | 66.7  (58.3-95.9) | 66.7  (56.2-77.1) | 0.530 | 66.7  (58.3-97.9) | 58.3  (50.0-75.0) | 0.174 | 66.7  (50.0-91.7) | 62.5  (58.3-87.5) | 0.810 | 66.7  (50.0-91.7) | 62.5  (58.3-87.5) | 0.810 |
| **Social Functioning** | 44.4  (11.1-66.7) | 27.8  (0.0-55.6) | 0.372 | 50.0  (33.3-66.7) | 22.2  (0.0-44.4) | 0.022 | 33.3  (0.0-55.6) | 38.9  (22.2-66.7) | 0.316 | 33.3  (0.0-55.6) | 38.9  (13.9-66.7) | 0.367 |
| **Health Perspective** | 50.0  (29.2-70.9) | 50.0  (25.0-58.3) | 0.322 | 54.2  (35.4-72.9) | 50.0  (25.0-66.7) | 0.202 | 50.0  (25.0-75.0) | 50.0  (25.0-66.7) | 0.735 | 50.0  (25.0-66.7) | 50.0  (25.0-66.7) | 0.842 |
| **Upper Resp. Symptoms** | 58.3  (45.9-83.3) | 62.5  (39.6-77.1) | 0.84 | 66.7  (52.1-83.3) | 58.3  (33.3-66.7) | 0.042 | 58.3  (50.0-66.7) | 58.3  (41.7-83.3) | 0.905 | 58.3  (41.7-66.7) | 58.30  (43.8-83.3) | 0.661 |
| **Lower Resp. Symptoms** | 66.7  (52.8-75.0) | 61.1  (37.5-68.1) | 0.251 | 63.9  (45.8-76.4) | 61.1  (33.3-66.7) | 0.218 | 50.0  (38.9-66.7) | 66.7  (56.9-77.8) | 0.071 | 55.6  (38.9-66.7) | 66.7  (51.4-77.8) | 0.112 |
| **Hearing Symptoms** | 66.7  (41.7-100.0) | 83.3  (66.7-100.0) | 0.105 | 83.3  (66.7-100.0) | 66.7  (50.0-100.0) | 0.068 | 66.7  (66.7-100.0) | 75.0  (54.2-100.0) | 0.967 | 66.7  (66.7-100.0) | 75.0  (54.2-100.0) | 0.967 |
